# Assessing self-reported public health emergency competencies for civil aviation personnel in China: a pilot study

**DOI:** 10.1186/s12889-024-18846-7

**Published:** 2024-07-28

**Authors:** Zuokun Liu, Yixin Li, Zhuo Li, Jingya Dong, Huan Yu, Hui Yin

**Affiliations:** 1 Department of Global Health, School of Public Health, Peking University Health Science Center, 38 Xue Yuan Road, Haidian District, Beijing, 100191 China; 2 Department of Epidemiology and Biostatistics, School of Public Health, Peking University Health Science Center, Beijing, 100191 China; 3 Institute of Area Studies, Peking University, Beijing, 100871 China; 4 Academy for Advanced Interdisciplinary Studies, Peking University, Beijing, 100871 China; 5 Institute of Global Health, Peking University Health Science Center, Beijing, 100191 China; 6 Center for Global Biosecurity Governance Research, China Foreign Affairs University, Beijing, 100037 China

**Keywords:** Aviation, Public health emergency, Preparedness, Response, Competency

## Abstract

**Introduction:**

COVID-19 has demonstrated the importance of competent staff with expertise in public health emergency preparedness and response in the civil aviation system. The civil aviation system is a critical sentinel and checkpoint to prevent imported cases and slow the spread of communicable diseases. Understanding the current competencies of staff to deal with public health emergencies will help government agencies develop targeted training and evidence-based policies to improve their public health preparedness and response capabilities.

**Methods:**

This cross-sectional pilot study was conducted from November 2022 to October 2023, involving 118 staff members from various positions within China’s civil aviation system. A 59-item questionnaire was translated and developed according to a competency profile. Data were collected using the self-report questionnaire to measure the workforce’s self-perceptions of knowledge and skills associated with public health emergency proficiency, categorized into (1) general competency, (2) preparedness competency, (3) response competency, and (4) recovery competency. KMO & Bartlett test and Cronbach’s α reliability analysis were used to test the reliability and validity of the questionnaire. Descriptive statistics, independent sample T-test, ANOVA, and linear regression models were performed to analyze the competencies.

**Results:**

A total of 107 staff members from the aviation system were surveyed in this study. The KMO & Bartlett test, (KMO = 0.919, *P* < 0.001) and Cronbach’s α coefficients (α = 0.985) for this questionnaire were acceptable. The results suggested that respondents scored a mean of 6.48 out of 9 for the single question. However, the staff needed to acquire more knowledge in investigating epidemic information (5.92) and case managing (5.91) in the response stage. Overall, males scored higher (409.05 ± 81.39) than females (367.99 ± 84.97), with scores in the medical department (445.67 ± 72.01) higher than management (387.00 ± 70.87) and general department (362.32 ± 86.93). Additionally, those with completely subjective evaluation (425.79 ± 88.10) scored higher than the general group (374.39 ± 79.91). To predict the total score, female medical workers were more likely to have lower scores (β = -34.5, *P* = 0.041). Compared with those in the medical department, the management workers (β = -65.54, *P* = 0.008) and general workers (β = -78.06, *P* < 0.001) were associated with a lower total score.

**Conclusions:**

There was still a gap between the public health emergency competencies of the civil aviation system and the demand. Staff in China’s civil aviation systems demonstrated overall competence in public health emergency preparedness and response. However, there was a need to enhance the accumulation of practical experience. Implementing effective training programs for public health emergencies was recommended to mitigate knowledge gaps. Meanwhile, regular training evaluations were also recommended to give comprehensive feedback on the value of the training programs.

## Introduction

The wide spread of the COVID-19 pandemic around the world had brought increased attention to the link between air travel and the spread of public health emergency. Air travel played an important role in the pandemic by allowing the virus to spread across the oceans and borders between continents at a much faster rate than in any previous era [1, 2]. According to Article 43 of the *International Health Regulations* (IHR, 2005), in the event of a Public Health Emergency of International Concern (PHEIC), the “Contracting States” could impose “Travel and Trade Restriction Measures” on the entry of passengers, goods, containers, depending on the spread, proliferation, and danger of communicable disease. In addition, Articles 25 and 28 of the regulations also stipulated specific provisions on aviation-related hygiene measures to provide a reference for specific aviation hygiene [3]. *Convention on International Civil Aviation* (1944. Article 14 of Chicago) also contained vague items relating to air transport restrictions on communicable diseases [4]. However, as an obstruction to international cooperation, travel restrictions violated the IHR, partly leading to countries’ hesitancy and dispute on aviation measures when the pandemic emerged [5]. Therefore the proper implementation of the airline response measures, and the high level of public health emergency competency among airline staff were of great importance in slowing down the spread of pathogens and preventing outbreaks.

Air travel played a significant role in promoting the spread of the epidemic. Airports are bustling hubs where domestic and international passengers frequently transit, necessitating stringent measures to prevent physical contact from serving as avenues for disease transmission. An analysis of the relationship between the aviation system and the prevalence of COVID-19 suggested that countries with more flight frequency and airports would have significantly higher infection rates [6]. Other studies evaluated travel restrictions in the 2009 H1N1 and 2019 COVID-19 pandemic, demonstrating the role of travel restrictions in reducing the international spread of communicable diseases [7, 8]. Measures like implementing “circuit breakers” (When the number of passengers testing positive for nucleic acid on a flight reaches a threshold, these air routes will be temporarily suspended) and restricting the number of flights have proven effective in reducing both the number of COVID-19 cases and the speed of transmission among patients and carriers [9]. . On the contrary, restrictive measures would significantly reduce flight frequency, ultimately resulting in incalculable losses in profitability [10]. The International Civil Aviation Organization (ICAO) estimated that the global international air passenger capacity in 2020 was 60% lower than in 2019 [11]. This resulted in a severe financial crisis, with most airlines grounded in the first half of 2020. The International Air Transport Association (IATA) estimated that the aviation industry would incur losses of $770 billion within six months, with total losses for airlines worldwide in 2020 estimated at $2.41 trillion [12]. There was a close relationship between the civil aviation system, the spread and prevention of the epidemic, and the sharing of weal and woe.

In the public health emergency mechanism of the civil aviation system, the comprehensive competency of personnel to respond to outbreaks played a key role, which meant the measurement and inspection of their competencies were essential prerequisites for the subsequent training and promotion [13]. However, there has been no established questionnaire for the personnel of the aviation system in China. Most of these investigations were focused on healthcare professionals, such as doctors or nurses [14, 15]. Moreover, because of the professionalism and particularity of the aviation system, the general medical system competency questionnaire may only be partially applicable.

This study primarily referenced a capability index system developed by the Netherlands National Centre for Infectious Diseases. This system is mainly based on the 4R theory of crisis management, proposed by Robert Heath in the book “Crisis Management,” which consists of four stages: Reduction, Readiness, Response, and Recovery. These stages correspond to various phases of emergency public health event prevention and control [16, 17]. Finally, this research expected to develop and compile a questionnaire on the competency of civil aviation system personnel in dealing with epidemics in China based on the existing index system [18] and distributed the questionnaire in different departments for reliability testing and preliminary application to assess the competency profiles of civil aviation system personnel in different dimensions and made corresponding recommendations.

## Methods

### The Chinese translation and revision of the questionnaire

The Chinese translation and revision of the questionnaire were divided into two stages. The former was based on translating a 59-item profile of communicable disease preparedness and response professionals in the air transport public health sector [18]. A preliminary questionnaire was prepared by using the 9-point Likert method according to the 59 competence items. In the second stage, the questionnaire was sent to experts from the Chinese Center for Disease Control and Prevention (CDC), China Entry-Exit Inspection and Quarantine (CIQ), colleges and customs, to obtain feedback opinions, and the questionnaire was revised based on the proposed advice to make it more suitable for Chinese civil aviation system. This questionnaire was based on the framework of 4R crisis management theory and was divided into four sections. The first section replaces “Reduction” with “General competency” since this index system only corresponds to manpower. The subsequent three sections of the questionnaire remain consistent with the 4R theory, including preparedness, response, and recovery stages.

### Preliminary study

Currently, there were no published studies utilizing this questionnaire or similar instruments to establish reference values. This was due to the fact that the questionnaire employed in this survey was newly developed in 2020 under the backdrop of the COVID-19 pandemic. Therefore, this study could only conduct preliminary study to estimate the sample size.

The translated questionnaire was distributed to 30 employees across various departments within the civil aviation system, with 10 randomly selected from each of the medical, administrative, and general departments. Upon analyzing the scores of these three groups of questionnaires, the mean and standard deviation were calculated for each group: medical (453.1 ± 72.8), administrative (399.6 ± 75.3), and general (386.7 ± 66.0), respectively. Using one-way analysis of variance F-tests from PASS 15, with α and β values set at 0.05 and 0.1, the estimated total sample size for this study was finally determined to be 90 people. Taking into account about 10% of invalid questionnaires, it was roughly estimated that this study required a sample of at least about 100 people.

#### Questionnaire distribution and data analysis

The questionnaire was distributed to civil aviation system personnel through the We Chat by using the electronic questionnaire platform, “Sojump”. A total of 118 questionnaires were obtained, of which 107 were finally included, after excluding the questionnaires with short response time, confusing logic, and consistent options. High-low grouping analysis [19, 20], KMO & Bartlett test [21], and Cronbach’s α test were used for questionnaire validity testing [22]. In this study, since only intergroup comparisons are involved, Cronbach’s alpha was set at a minimum value of 0.7. Questionnaire items with Cronbach’s α below this threshold will be removed [23]. Descriptive analysis was used to illustrate the respondents’ basic information. T-tests and variance analysis were used to test the differences. Linear regression models were finally constructed by selecting factors of influence. SPSS 26.0 and R 4.2.3 were used as data analysis software.

## Results

### Model of public health emergency competencies and questionnaire framework

**Fig. 1:**
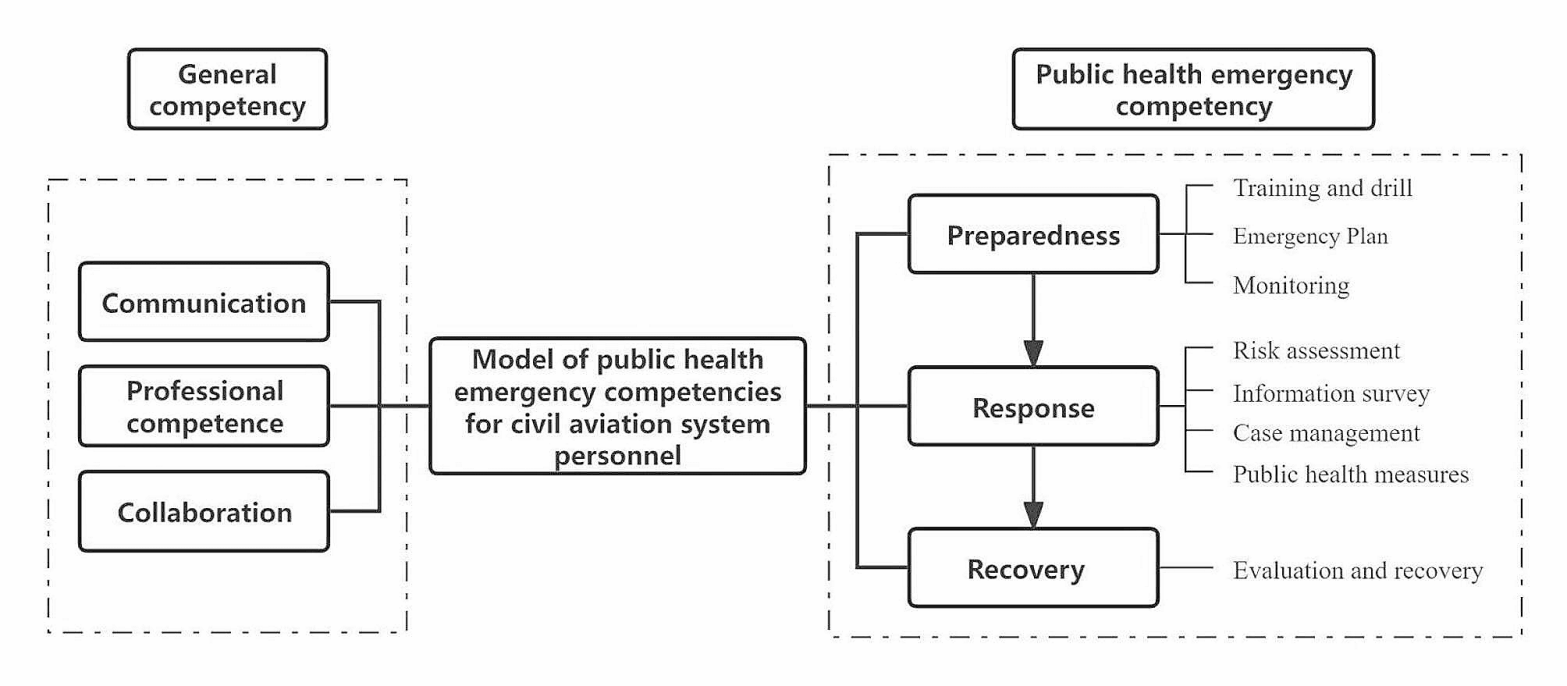
Model of public health emergency competencies for civil aviation system personnel

After Chinese translation and modification, as Fig. 1 shows, a model of public health emergence competencies for civil aviation system personnel was established. The model contained two main dimensions, including “General competency” and “Public health emergency competency”, and six main competencies, including “Communication”, “Professional competence”, “Collaboration”, “Preparedness”, “Response” and “Recovery”. Table 1 indicates the description of the six competencies. The three competencies under the category of public health emergency were subdivided into eight more detailed competency indicators, so there were a total of 11 competency indicators that could be investigated. Based on that, the questionnaire containing 64 questions was established and distributed, of which five were for basic information, and 59 were for evaluating competencies.

**Table 1:** Description of public health emergency competencies for civil aviation system personnel

Competency		Description
General competency	Communication	Understanding the basic principles of risk communication, mastering effective communication methods and channels, and knowing relevant terminology.
	Professional competence	Knowledge of basic public health knowledge, familiarity with specific public health measures
	Collaboration	Clarify team positioning and responsibilities to implement established plans by achieving multi-disciplinary and multi-department cooperation.
Public health emergency competency	Preparedness	Conduct training and drills in daily period, develop a detailed emergency response plan, and implement health monitoring.
	Response	Implement risk assessment, carry out epidemiological information surveys, manage and control cases and contacts, and implement prevention and control measures.
	Recovery	Evaluate and summarize the emergency process, update the original plan based on the emergency, and recover the aviation system as soon as possible once the outbreak was controlled or alleviated.

### Validity and reliability of the questionnaires

The total and individual stage scores were analyzed by high-low grouping analysis (Table 2), which taking the top 27% and the bottom 27% scores and dividing them into two groups for an independent sample t-test [19]. The results all met the significance criteria and showed a statistical difference, indicating good questionnaire validity.

**Table 2:** Validity analysis results of each item in the questionnaire

	Grouping	Mean ± SD	t	*P*
Communication	1	15.83 ± 3.53	14.627	< 0.001***
	2	25.79 ± 1.01		
Professional competence	1	10.52 ± 2.23	15.07	< 0.001***
	2	17.21 ± 0.86		
Collaboration	1	26.45 ± 3.81	12.784	< 0.001***
	2	35.62 ± 0.62		
Training and drill	1	16.93 ± 2.65	16.806	< 0.001***
	2	25.86 ± 1.10		
Emergency plan	1	43.03 ± 7.38	18.936	< 0.001***
	2	73.72 ± 4.67		
Monitoring	1	21.07 ± 4.74	19.579	< 0.001***
	2	40.76 ± 2.61		
Risk assessment	1	25.97 ± 6.52	18.989	< 0.001***
	2	50.9 ± 2.73		
Information survey	1	24.76 ± 10.51	19.229	< 0.001***
	2	65.55 ± 4.49		
Case management	1	18.52 ± 6.75	22.238	< 0.001***
	2	49.76 ± 3.41		
Public health measures	1	34.03 ± 9.36	21.778	< 0.001***
	2	75.59 ± 4.25		
Evaluation and recovery	1	16.45 ± 5.65	16.276	< 0.001***
	2	34.24 ± 1.64		

The KMO and Bartletts chi-square test results (Table 3) showed that the items were suitable for factor analysis (KMO = 0.919, *P* < 0.001). Cronbach’s α reliability analysis of the questionnaire showed that the overall Cronbach’s α coefficient was 0.985, and Cronbach’s α coefficients of the four stages were 0.928, 0.952, 0.983, and 0.929. Respectively, all coefficients were more significant than 0.70. Indeed, the majority of items had α values greater than 0.9. The results indicated that the questionnaire reliability was acceptable, so no items were removed.

**Table 3:** Questionnaire reliability analysis results

Dimension			Cronbach’s α coefficient
General competence			0.928
		Communication	0.855
		Professional competence	0.762
		Collaboration	0.897
Preparedness			0.952
		Training and drill	0.901
		Emergency Plan	0.924
		Monitoring	0.875
Response			0.983
		Risk Assessment	0.934
		Information survey	0.964
		Case management	0.945
		Public health measures	0.943
Recovery			0.929
		Evaluation and recovery	0.929
Overall scale			0.985

### Results of public health emergency competencies of personnel in civil aviation systems

#### Basic information

As shown in Table 4, a total of 107 staff members from the aviation system were surveyed in this study, with 69 (64.5%) females and 38 (35.5%) males. All departments were divided into three sorts, including medical, management, and general posts. Among them, medical departments mainly included health management departments or medical centers in the civil aviation system (18 persons, 16.8%). The management departments mainly included civil aviation bureaus or local administrations (27 persons, 25.2%). The general posts were the majority and mainly included front-line airport workers (56 persons, 57.9%). The length of service was stratified from less than five years to more than 15 years and distributed uniformly in amount. Subjective evaluation refers to the overall subjective evaluation of the respondent’s competency to prevent and control epidemics. Most respondents thought that they were completely or basically competent, and only four people thought that they had difficulty meeting the demands of a public health emergency.

**Table 4:** Basic information of the investigated personnel

Basic Information		*n*	%
Sex	Male	38	35.5
	Female	69	64.5
Department	Medical Department	18	16.8
	Management Department	27	25.2
	General post	62	57.9
Length of service	< 5 years	32	29.9
	5–14 years	49	45.7
	≥ 15 years	26	24.2
Subjective evaluation	Be not competent	4	3.7
	Basically competent	79	73.8
	Completely competent	24	22.4

- Scores of civil aviation system personnel’s public health emergency competencies.

As Table 5 shows, The values displayed show the mean scores of all staff in different epidemic stages, due to varying numbers of questions in each stage, the scores across stages weren’t directly comparable. Therefore, mean scores were calculated for each stage by dividing the total score by the number of questions in that stage and then computing the mean, which were all capped at 9 points. The statistical analysis revealed that respondents scored a mean of 6.48 for the total questionnaire. The respondents scored high in essential general competencies, preparedness and recovery phases but performed poorly in the response stage. On a detailed scale, the three general competencies were all scored ≥ 7. In the stages of epidemic prevention, the training and drills in the preparation stage got a high score of 7.26, indicating the adequacy of daily training. However, the lowest scores for investigating epidemic information (5.92) and case managing (5.91) were in the response stage. On the whole, the score reflected the relative insufficiency of personnel competencies in the actual epidemic response activities and the implementation of measures.

**Table 5:** Scores of each public health emergency competencies item

Category		Mean scores	Score ≥ 7 (%)
General competence		7.37 ± 1.12	65.40
	Communication	7.01 ± 1.44	54.20
	Professional competence	7.07 ± 1.43	64.50
	Collaboration	7.78 ± 1.01	84.10
Preparedness		6.60 ± 1.33	43.00
	Training and drill	7.26 ± 1.25	69.20
	Emergency plan	6.53 ± 1.40	43.00
	Monitoring	6.33 ± 1.63	43.90
Response		6.14 ± 1.85	38.30
	Risk assessment	6.59 ± 1.70	49.50
	Information survey	5.92 ± 2.09	36.40
	Case management	5.91 ± 2.13	38.30
	Public health measures	6.21 ± 1.87	39.30
Recovery		6.49 ± 1.87	45.80
	Evaluation and recovery	6.49 ± 1.87	45.80
Total Score		6.48 ± 1.45	38.30

*Note*: The values displayed showing the scores of all staff in different epidemic stages and their total score (Mean + SD)

**Fig. 2:**
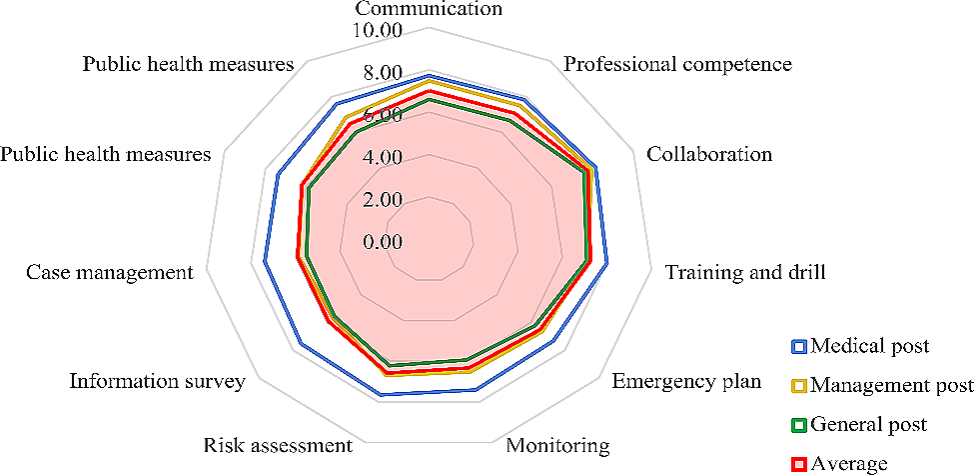
Radar chart of distribution of public health emergency competencies in different positions

According to the different position types, the radar chart of the ability distribution was drawn (Fig. 2). There were obvious differences among the three different position types. Medical position (blue line) had highest score in all the competencies. In contrast, the competencies of management staff (yellow line) were almost identical to the average (red line). The personnel in general posts (green line) were generally lower than average.

b) Analysis of the variability of personnel competencies in civil aviation systems.

The results of the difference test show that gender, type of occupation, and level of subjective evaluation have statistical significance on the competence score except for the length of service (Table 6). Overall, males scored higher (409.05 ± 81.39) than females (367.99 ± 84.97), with scores in the medical department (445.67 ± 72.01) notably higher than those in the management (387.00 ± 70.87) and general department (362.32 ± 86.93). Additionally, those with completely subjective evaluation (425.79 ± 88.10) scored higher than those in the general group (374.39 ± 79.91). There was a slight increase in general competency score by length of service, whereas there was no significant statistical difference. Three statistically significant influencing factors were selected, and a multiple linear regression equation was used to establish a model to predict the total score (Table 7). There was no significant difference between male and female staff in general competencies. However, male staff scored higher at three public health emergency stages. As a whole, female medical workers were more likely to have lower scores (β = -34.5, *P* = 0.041). Compared with those in the medicine department, the management workers (β = -65.54, *P* = 0.008) and general workers (β = -78.06, *P* < 0.001) were associated with a lower total score. In addition, those who rated their overall subjective evaluation better had higher competence significantly than those who were lower in all stages. Workers with completely subjective evaluation were likelier to have higher scores (β = 36.7, *P* = 0.054) than workers with basically competent.

**Table 6:** Impact of factors on public health emergency competencies of civil aviation system personnel

Characteristic		*n*	General competence	Preparedness	Response	Recovery	Total Score
**Sex**							
	Male	38	68.16 ± 8.94	118.21 ± 20.71	194.63 ± 49.90	28.05 ± 6.7	409.05 ± 81.39
	Female	69	65.26 ± 10.58	108.9 ± 22.95	169.04 ± 53.64	24.78 ± 7.68	367.99 ± 84.97
	P-value		0.156	0.040*	0.017*	0.030*	0.017*
**Length of service**							
	< 5 years	32	64.38 ± 9.85	111.97 ± 21.43	180.59 ± 49.20	25.50 ± 6.22	382.44 ± 82.03
	5–15 years	29	66.10 ± 8.75	110.80 ± 21.45	179.94 ± 51.77	26.29 ± 7.29	383.12 ± 82.14
	>=15 years	18	69.00 ± 12.29	115.15 ± 26.21	171.69 ± 62.84	25.85 ± 9.35	381.69 ± 98.96
	P-value		0.219	0.730	0.782	0.898	0.998
**Department**							
	Medical	18	71.44 ± 8.16	127.00 ± 21.87	216.83 ± 39.55	30.39 ± 5.51	445.67 ± 72.01
	Management	27	69.26 ± 7.80	113.96 ± 17.61	176.41 ± 49.48	27.37 ± 6.30	387.00 ± 70.87
	General	62	63.5 ± 10.60	107.15 ± 22.91	167.65 ± 54.28	24.03 ± 7.85	362.32 ± 86.93
	P-value		0.002**	0.003**	0.002**	0.003**	0.001**
**Subjective evaluation**							
	Basically competent	79	64.89 ± 9.43	109.73 ± 22.01	174.32 ± 49.55	25.46 ± 6.91	374.39 ± 79.91
	Completely competent	24	72.75 ± 8.26	124.46 ± 19.89	199.67 ± 60.25	28.92 ± 8.30	425.79 ± 88.10
	P-value		< 0.001***	0.004**	0.040*	0.043*	0.008**

*Note* *P003C0.05;**P003C0.01;***P003C0.001; The values displayed showing scores of each group at different epidemic stages and their total score(Mean + SD)

**Table 7:** Linear regression models predicting the total Scores

Characteristic	Estimate	t value	*P*
**Sex**			
Male			
Female	-34.54	-2.065	0.041*
**Department**			
Medical			
Management	-65.54	-2.711	0.008**
General	-78.06	-3.718	0.000***
**Subjective evaluation**			
Basically competent			
Completely competent	36.7	1.952	0.054

Note*P003C0.05; **P003C0.01; ***P003C0.001 .

## Discussion

This study developed a model and questionnaire of public health emergency competencies for civil aviation personnel. The terminology and scenarios used in the questionnaire were aligned with the actual situation of the civil aviation personnel’s work. After modification, the final analysis showed high reliability and validity. This indicated that the questionnaire’s quality and translation were acceptable, meeting the professional skills of the surveyed civil aviation staff.

The questionnaire was divided into various dimensions according to different phases of the epidemic. Overall, the competencies of civil aviation system personnel scored moderately, with room for improvement in some items, especially in the response stage. Most civil aviation personnel had an acceptable level of competence, which meant that they could meet the basic needs of the civil aviation system to ensure regular operation during public health emergencies. The relatively high general competency scores showed an intention to collaborate and the basic professional skills required to implement outbreak control. However, the staff needed training to become more skilled in policies and response, to deal with complex, uncertain epidemic emergency in the actual response process. In the subsequent training, more emphasis also needed to be placed on practical effects. In fact, studies have shown that simulation drills can help both medical and aviation personnel improve their ability to respond to epidemic or accidents [24, 25].

Various factors, including gender, position, and subjective evaluation, crucially influenced the final scores. Males were comparable to females in general competencies but had higher scores in each public health emergency stage, presumably because they were inborn open-minded, rational and calmer in emergencies [26]. It was not surprising that medical personnel, with their professional knowledge and skills, were more likely to be exposed to actual outbreaks, face patients and have a fairly strong competitive advantage in dealing with public health emergence. Furthermore, those with optimistic subjective evaluation also had higher final scores on the questionnaire, which indicated that self-confidence and optimism were more beneficial in dealing with public health emergencies, which had similar results in other studies [27, 28]. Length of service did not affect public health emergency competencies, possibly because their daily work experience was not directly related to experiencing a significant epidemic over a long period. These results were similar to those of previous studies on the competency of health system personnel [14, 15].

Some recommendations were made about the model of public health emergency competencies for civil aviation system personnel. Primarily, the epidemic information acquisition and public health emergency treatment in the emergency stage were the top priorities that needed urgent improvement. Therefore, in later training, emphasis should be placed on simulating responses in real emergencies to increase familiarity and understanding of on-site emergency treatment [29]. Then, the post differences were crucial factors affecting civil aviation personnel’s competencies. Generally, the front-line workers were less capable of responding to public health emergencies. However, they were often exposed to people infected with communicable diseases in real situations. Therefore, more specific training was required to better serve as the first barrier in the face of public health emergencies.

## Limitations

The questionnaire was developed based on an existing English aviation system competency model of the public health emergency. Though carefully translated, there were still differences between the expressions and idioms, making the questionnaire challenging for respondents. Secondly, although all items in this questionnaire had Cronbach’s α coefficients greater than 0.7, a few of them exceeded 0.95, indicating a probable high level of content consistency among the questionnaire items [30]. In addition, because of the lack of similar prior studies, the estimate of the sample size of civil aviation staff might be below the actual requirement due to the bias of preliminary survey. The proportion of each unit and occupation type needed to be balanced. The number of medical and management staff was lower, which is related to the difference in the proportion of the number of positions in the civil aviation system. The above questions indicated that the questionnaire still needed to be mature. Subsequent linguistic refinements and improvements to the questionnaire itself were needed. There were still problems with the survey process, and subsequent studies with more adequate sample sizes were pending.

## Conclusion

This study developed a localized competency questionnaire based on the competency model developed by the Dutch CDC for preventing and controlling outbreaks in the aviation system. A reliability test and preliminary application study were conducted. According to the results, the questionnaire was usable. However, the public health emergency competencies to prevent and control the epidemic were weak. The stage of response phase for civil aviation staff, gender, position, and subjective evaluation would crucially influence the competence. The Chinese questionnaire can theoretically be used to investigate the public health emergency competencies of personnel in the civil aviation system. Researchers can analyze the scoring characteristics of different departments, age groups, or other demographics. New training and improvement plans can be formulated based on data analysis results.

## Data Availability

Availability of data and materialsThe datasets used and/or analysed during the current study are available from the corresponding author on reasonable request.

## Abbreviations

WHO: World Health Organization
PHEIC: Public Health Emergency of International Concern
IHR: International Health Regulations
CDC: Centers for Disease Control and Prevention (Chinese)
ICAO: International Civil Aviation Organization
CIQ: China Entry-Exit Inspection and quarantine
KMO: Kaiser-Meyer-Olkin
COVID-19: Coronavirus disease 2019
IATA: International Air Transport Association
